# Coinfection of *Toxoplasma gondii* and Other Microorganisms: A Systematic Review and Meta-Analysis

**DOI:** 10.3390/microorganisms12102106

**Published:** 2024-10-21

**Authors:** María de la Luz Galván-Ramírez, Eber Eduardo Soto-Hernández, Rocío Bojórquez-Pérez

**Affiliations:** 1 Departamento de Microbiología y Patología, Centro Universitario de Ciencias de la Salud, Universidad de Guadalajara, Guadalajara 44340, Jalisco, Mexico; eber.soto9869@alumnos.udg.mx; 2 Escuela de Biología, Universidad Autónoma de Sinaloa, Culiacán 80050, Sinaloa, Mexico; rociobojorquez15@gmail.com

**Keywords:** coinfection, toxoplasmosis, parasites, virus, bacteria

## Abstract

Toxoplasmosis is a disease caused by the intracellular protozoan *Toxoplasma gondii*, which has infected a third of the global population. Immunocompromised individuals and children with congenital disorders are most likely to be impacted by toxoplasmosis, and accurate diagnosis is essential. Toxoplasmosis is associated with HIV, schizophrenia, and diabetes. However, few studies have analyzed the association with other microorganisms. The purpose of this study was to determine the prevalence of coinfection of *Toxoplasma gondii* with other pathogens. From November 1997 to June 2024, PubMed, Science Direct, LAT index, Web of Science, Google Scholar, and Research Gate were searched. The keywords used were “*Toxoplasma* and microorganism coinfection”, “*Toxoplasma* coinfection and parasites”, “*Toxoplasma* coinfection and Protozoans or Bacteria or Helminths or Nematodes or Trematodes or Mycobacterium”, “*Toxoplasma gondii* in coinfection with virus”, and “Human Toxoplasmosis and coinfection”. Next, OpenMeta Analyst Software version 12.11 was used for meta-analysis, creating forest plots, and determining heterogeneity I^2^. A total of 17,535 patients in 48 articles, of whom 5848 were seropositive to *T. gondii*, were included in this review. Population studies showed that the prevalence of virus infection was most frequent (32%), followed by parasites (18.4%), bacteria (29.7%), and fungi (5.8%). The pooled prevalence of coinfection was found to be 29.1%, with a lower bound of 0.232, an upper bound of 0.350, a standard error of 0.030, and *p* < 0.001. Heterogeneity (I^2^) was 99.12%, *p* < 0.001, with a global variance tau2 = 0.042. *Toxoplasma gondii* is an opportunist that mainly affects immunocompromised populations. The main coinfections were found to be viral infections, with HIV ranking first, followed by cytomegalovirus, hepatitis B and C, rubella, herpes simplex 1 and 2, SARS-CoV-2, and coxsackie virus.

## 1. Introduction

Toxoplasmosis is a zoonotic disease affecting animals and humans that is caused by the obligate intracellular protozoan *Toxoplasma gondii* (*T. gondii*). *Toxoplasma* has a large number of intermediate hosts as it infects humans and a wide variety of animals of different vertebrate species; these include rodents, marsupials, insectivores, primates, lagomorphs, and birds. However, the most representative species are found in the feline family; these include pumas, lynxes, jaguars, and cats that live in close proximity to human populations. The dynamics of infection and the wide spread of the disease are due to the high resistance of oocysts in the environment. Sexual reproduction of *Toxoplasma* takes place in felines, which discard millions of oocysts that then contaminate fruits and vegetables. *T. gondii* also reproduces asexually in intermediate hosts in both animals and humans. People who live with cats face a higher risk of transmission because an infected cat can excrete up to 20 million oocysts, after 3–10 days postinfection of cysts in tissues [1,2,3].

*Toxoplasma* presents three forms according to infection status. Tachyzoites are responsible for the acute phase of the infection. Bradyzoites are present in tissue cysts, the brain, and skeletal muscles, as well as the sporozoites in oocysts. After entering the host, bradyzoites and sporozoites transform into tachyzoites within tissues. Cysts can remain dormant during the life of the host only if the immune system is in balance [1,3,4].

The transmission mechanisms are as follows: orally through ingesting food contaminated with oocysts and cysts, especially raw or undercooked meat and organ transplants. Tachyzoites are transmitted transplacentally and via blood transfusion and directly when existing hand injuries of workers in laboratories, butcher shops, and slaughterhouses [1,3,4]. Furthermore, transmission via ingestion of undercooked meat is more prevalent in populations where such meat is part of the diet.

In one study conducted in the USA, researchers quantitatively examined the microbial risk posed by domestically produced lamb; specifically, the prevalence of *T. gondii* in lambs that went to market in 2011 was determined. The bradyzoite concentration in cysts was determined using log-linear regression and an exponential dose–response model. The authors found the average probability of *Toxoplasma* infection per portion of lamb consumed; this suggested approximately 6300 new infections per year in the American population. Immunocompromised individuals and pregnant women are most seriously impacted by toxoplasmosis [1,4,5].

Regarding climatic parameters, the highest prevalence rates for toxoplasmosis have been recorded in regions with a mean relative humidity of 80% (46.6%), annual precipitation of 1000–1500 mm (39.2%), and mean annual temperature of 30 °C (36.5%), with lower rates being recorded in regions with humidity < 40% (27.0%) and annual precipitation of 250–500 mm (26.8%).

Approximately one-third of the global population has been infected with the parasite [1,4]. However, acquired infection can be asymptomatic in immunocompetent individuals, who usually do not know they are infected. However, some people exhibit flu-like symptoms, including fever, lymphadenopathy that may persist for weeks, headaches, muscle aches, and skin rashes.

Ocular toxoplasmosis can present as eye pain, poor vision, and floaters. If such eye disease is untreated, it can cause severe chorioretinitis and vision loss [1]. Ocular disease is more severe in people with immunodeficiency [1].

Toxoplasmosis may also affect the brain. The apparent disappearance of the parasite in some organs may be due to a protective immunologic response, but this is not the case when it is in the Central Nervous System (CNS), where the persistence and proliferation of *T. gondii* is due to the limited spread of antibodies to this tissue. When this occurs, an area of periventricular alterations can be observed locally, delimited by cellular infiltrates and thrombosis of small and large vessels, with varying degrees of necrosis. In addition, there is intense multiplication of microglia cells, death of astrocytes, and formation of lymphocytic and granulocytic infiltrates, as well as the presence of fibrous material that affects nearby capillaries. The clinical manifestations of subacute toxoplasmosis may be associated with hydrocephalus, seizures, as well as damage to the choroid [1].

The genome of *T. gondii* contains two genes encoding tyrosine hydroxylase; this produces levodopa (L-DOPA), a precursor to dopamine. The encoded enzymes metabolize phenylalanine and tyrosine. One of the genes of *Toxoplasma*, *TgAaaH1*, is constitutively expressed; in addition, the gene *TgAaaH2* is induced by bradyzoite formation during the life cycle of cyst formation. This interaction induces high levels of dopamine in brains infected with *Toxoplasma*, which can result in schizophrenic symptoms [1,3]. Since the beginning of the 21st century, several studies have associated chronic toxoplasmosis with schizophrenia [6]. Toxoplasmosis has also been associated with bipolar disorder and with certain behavioral problems [6]. Several epidemiological studies have shown high prevalence rates of anti-*Toxoplasma* antibodies in different countries, for example, Brazil (91.8%), Ethiopia and France (87.9%), Iran (42%), Tunisia (40%), Mexico (51.5%), and Egypt (31.75%).

Toxoplasmosis is mainly associated with immunocompromised individuals, including patients with HIV, AIDS, and cancer, as well as transplant recipients. Individuals in all of these groups face a high risk of *Toxoplasma* infection, especially if they already have a latent toxoplasmosis. According to a 2023 WHO report, approximately 88.4 million people have been infected with HIV, and around 42.3 million people have died from HIV-related causes. Globally, 39.9 million people were living with HIV at the end of 2023. The high prevalence of infected people increases the likelihood of numerous coinfections with *Toxoplasma*. It is important to note that if these patients are not diagnosed promptly, they can develop fatal encephalitic toxoplasmosis. A small number of studies have also been conducted on toxoplasmosis in cancer patients and transplant recipients [1,2,3]. However, HIV was reported as a comorbidity in 40% of toxoplasmosis cases and accounted for more than half of the direct healthcare costs associated with clinical toxoplasmosis.

Accurate diagnosis is essential in *T. gondii* infections, especially in immunocompromised individuals and those affected by congenital transmission. There is a wide variety of standardized serologic methods to detect the infection by using IgG, IgM, and IgA antibodies. Chronic infection is diagnosed by IgG antibodies, and acute infection by IgM antibodies [1], and IgA has been used to detect recent infections of pregnant women [1]. The enzyme-linked immunosorbent assay (ELISA) and real-time loop-mediated amplification polymerase chain reaction (RT- PCR/LAMP) methods may both be used for parasite detection in the umbilical cord blood of pregnant women with acute infection. In HIV patients, analysis of cerebrospinal fluid with RT-PCR/LAMP, magnetic resonance imaging, and computed tomography of the brain may all be used to obtain accurate diagnoses [4].

For infected pregnant women, treatment with spiramycin is associated with a low rate of side effects. After 16 weeks of pregnancy, pyrimethamine (PYR) treatment with sulfadiazine (SDZ) is typically prescribed; however, this teratogenic treatment should be avoided before the 14th week. In addition, PYR combined with SDZ and folic acid is suggested for immunocompromised patients. However, PYR is unavailable in many countries, so trimethoprim/sulfamethoxazole is typically the first treatment applied in these locations [1,5].

Currently, toxoplasmosis is associated with different microorganisms in case reports. The most frequently reported coinfections have been with viruses such as human immunodeficiency virus (HIV), cytomegalovirus (CMV), hepatitis C (HCV), and severe acute respiratory syndrome (SARS-CoV-2). Coinfections with tuberculosis (TB) and T*oxocara* have also been reported. Toxoplasmosis has also been reported to affect immune response and raise the risk of complications [2,3]. Therefore, it is clearly important that better knowledge be sought regarding toxoplasmosis and coinfection with other microorganisms.

Toxoplasmosis is associated with immunocompromised patients, as well as individuals affected by schizophrenia or diabetes. However, there have been few studies on the association of toxoplasmosis with other diseases. The purpose of this study was to determine the prevalence of coinfection of *Toxoplasma gondii* with other pathogens.

## 2. Materials and Methods

### 2.1. Main Search Strategy

**Figure 1:**
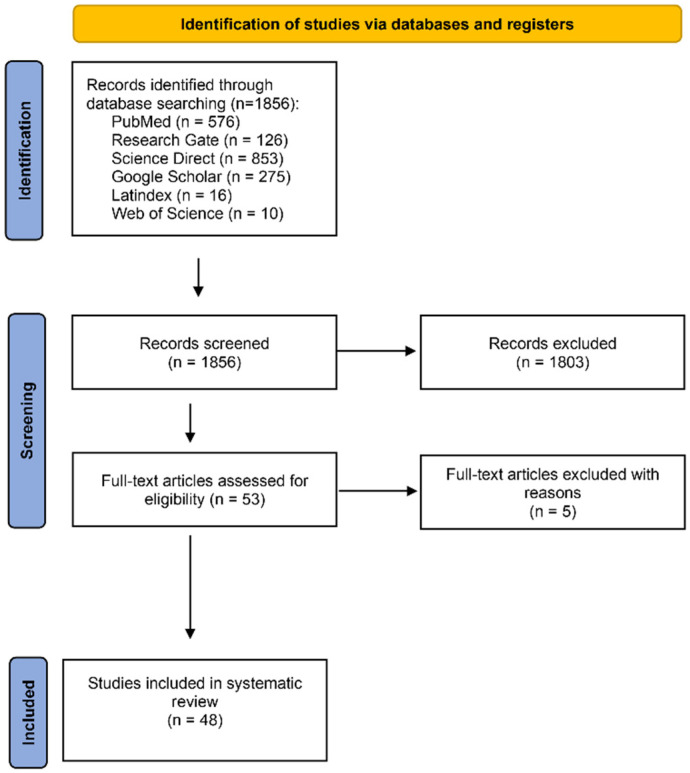
Search method flowchart, data collection, and database search.

This study followed the general methodology recommended for systematic reviews; this involved the use of a flowchart with the elements required for the data search, as illustrated in Figure 1 [7,8,9]. Six public databases (PubMed, Science Direct, Latin Index, Web of Science, Google Scholar, and Research Gate) were searched for articles published between November 1997 and June 2024. The keywords used were “*Toxoplasma* and microorganism coinfections, “*Toxoplasma* coinfections and parasites”, “*Toxoplasma* Coinfections and Protozoans or Bacteria or Helminths or Nematodes or Trematodes or Mycobacterium”, “*Toxoplasma gondii* in coinfection with virus”, and “Human Toxoplasmosis in coinfection”.

### 2.2. Data Collection

All articles were reviewed carefully by two investigators (G.R.M.L. and E.E. S. H.). The extracted data included the year of publication, design of studies, population, location, type of study, sample size, number of cases studied, number of positive results for *Toxoplasma* and other microorganisms, and diagnostic test used.

### 2.3. Inclusion Criteria

Inclusion criteria specified full texts of original articles in English in which humans were the object of study. Review articles, those with uncompleted information, and animal studies were all excluded.

Two reviewers independently reviewed all articles [10,11,12,13,14,15,16,17,18,19,20,21,22,23,24,25,26,27,28,29,30,31,32,33,34,35,36,37,38,39,40,41,42,43,44,45,46,47,48,49,50,51,52,53,54,55,56,57]. The search strategy is shown in Figure 1.

### 2.4. Meta-Analysis

Open Meta-Analyst version 12.11 for Windows 10 (Brown University, Providence, RI, USA) was used for all statistical tests.

The pooled prevalence of coinfection with *Toxoplasma gondii* and other microorganisms was estimated. A binary random-effect model was used for highly heterogeneous data (I^2^ > 50%). Calculations of heterogeneity (I^2^) for sums of individual groups or several subgroups were expressed in the form of percentage and significance (*p*).

### 2.5. Forest Plots

A model of random effects for summary statistics was applied to calculate the parameters of effect size and rate of occurrence with a confidence level of 95%. The estimate (pooled prevalence), expressed as a decimal fraction, was multiplied by 100% to obtain the prevalence value. For each study, high and low range limits and numbers of coinfection cases within the /N total were obtained.

## 3. Results

### 3.1. Study Selection

We found 1856 studies potentially related to *T. gondii* and coinfections. However, only 48 met the inclusion criteria for this systematic review and meta-analysis, as set out in Figure 1 [10,11,12,13,14,15,16,17,18,19,20,21,22,23,24,25,26,27,28,29,30,31,32,33,34,35,36,37,38,39,40,41,42,43,44,45,46,47,48,49,50,51,52,53,54,55,56,57]. Three categories were used for the purpose of our analysis: (A) general population studies on *Toxoplasma gondii* and its relationship with other coinfections; (B) cases and controls; and (C) subgroups of types of microorganisms (Table 1).

Details of symptoms, types of microorganisms, diagnostic methods, prevalence of anti-*Toxoplasma* antibodies, and treatments applied are specified in Table 2.

**Figure 2:**
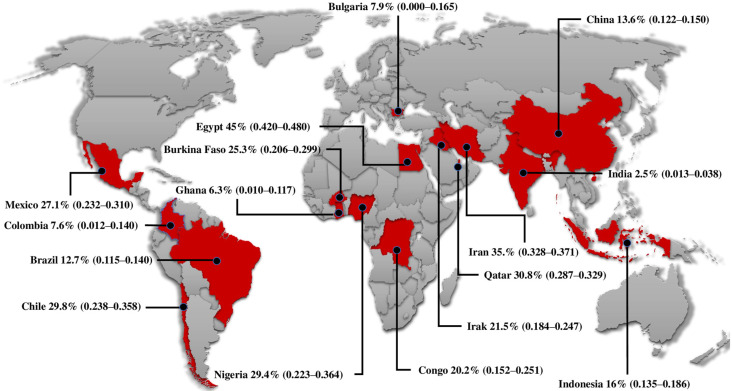
Pooled prevalence in patients with *Toxoplasma* infection and coinfection with other microorganisms in different countries. Prevalences are expressed as percentages (%) with upper and lower limits.

**Figure 3:**
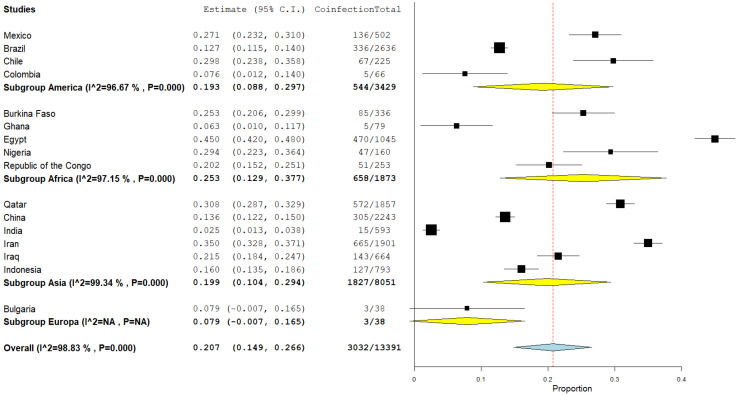
Prevalence of coinfection by continent. The black squares represent the effect size (estimate) with 95% C.I upper and lower bound (dashed lines) for each study; the yellow diamonds represent pooled prevalences in individual continents; the blue diamond represents global pooled prevalence; I^2=Heretogenity; =99.13%, (*p* < 0.001) (references [10,11,12,13,14,15,16,17,18,19,20,21,22,23,24,25,26,27,28,29,30,31,32,33,34,35,36,37,38,39,40,41,42,43,44,45,46,47,48,49,50,51,52,53,54,55,56,57]).

The selected articles were published in 16 different countries. Four studies were published in the Americas, five in sub-Saharan Africa, six in Asia, and one in Europe (Figure 2 and Figure 3).

The most frequently reported coinfection with *Toxoplasma gondii* was with human immunodeficiency virus (HIV); this was described in 14 studies (29.16%) involving a total of 936 coinfected patients. Coinfection with SARS-CoV-2 was described in 12 articles (25.0%), involving a total of 701 patients. Five population studies described coinfection with *hepatitis* (10.41%); one of these studies involved hepatitis B, while the remaining four involved hepatitis C; in all, 238 coinfected patients were described in these 5 works. With respect to *rubella* coinfection, 4 studies (8.33%) were identified, including populations of pregnant women and children, involving 608 patients in total. Four articles (8.33%) described coinfection with cytomegalovirus; 667 patients were involved in these studies. Three articles (6.25%) described coinfection with herpes simplex virus; 450 patients were involved in these studies. Finally, coinfection with *coxsackievirus* was described in 1 study (2.08%) in which 35 patients were involved. Regarding coinfection with parasites, *Toxocara* spp. was described in 6 studies (12.5%) involving a total of 255 patients. One study (2.08%), involving five patients, mentioned *Plasmodium falciparum*. Regarding coinfection with bacteria, 5 studies (10.41%), involving a total of 324 patients, mentioned *Mycobacterium tuberculosis*. Only 1 study (2.08%), involving 31 patients, mentioned *Helicobacter* p*ylori*. Coinfection with fungi was described in only two studies (4.16%), both of which involved *Pneumocystis jirovecii*, and included nine patients in total. Finally, with respect to multiple coinfections with viruses and bacteria, two studies (2.08%) were identified. One of these studies described *varicella zoster*, tuberculosis, and *cytomegalovirus*; the other described *Entamoeba histolytic*, *Schistosoma manssoni*, and virus hepatitis C. In all, 117 patients were involved in these 2 works.

### 3.2. General Prevalence Studies on Toxoplasma gondii and Its Relationship with Other Coinfections

**Figure 4:**
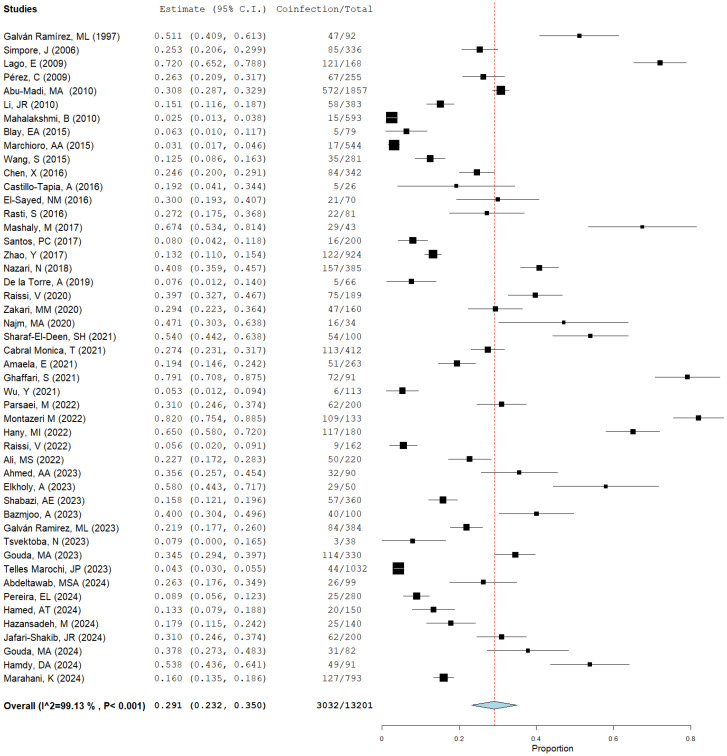
Forest plot of the prevalence of global *Toxoplasma* coinfection (29.1%). The blue diamond is the pooled prevalence, the black squares show size effects, and the lines represent confidence intervals. I^2=Heretogenity; NA: not applicable, (references [10,11,12,13,14,15,16,17,18,19,20,21,22,23,24,25,26,27,28,29,30,31,32,33,34,35,36,37,38,39,40,41,42,43,44,45,46,47,48,49,50,51,52,53,54,55,56,57]).

A total of 48 population studies were selected for the analysis and processing of data related to *Toxoplasma gondii* with coinfections of other microorganisms. The pooled prevalence of coinfection was 29.1%, with a lower bound of 0.232, an upper bound of 0.350, a standard error of 0.030, *p* < 0.001, I^2^ (99.12%, *p* < 0.001), and global variance (tau2) of 0.042 (Figure 4).

**Figure 5:**
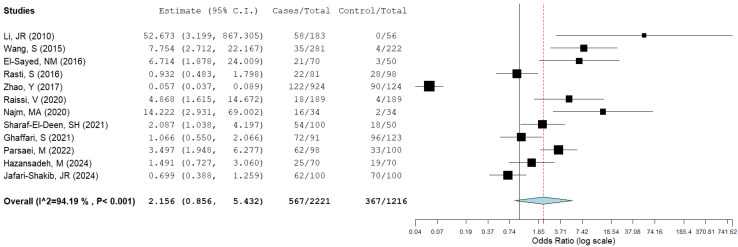
Meta-analysis of cases and controls. The blue diamond represents pooled prevalence, the I^2=Heretogenity= (94.1%), *p* > 0.001), the estimate of the risk is OR (black square) and there is a 95% CI for the upper and lower bounds (lines) (references [15,19,22,23,26,29,31,32,35,37,53,54]).

Twelve articles involved cases and controls analyzed with meta-analysis. Among these, heterogeneity was found to be 94.19%, which is lower than in the global analysis, with OR = 2.156 and 95% CI = 0.856–5.432. These articles described 2221 cases, involving 567 coinfected patients; out of 1216 controls, 367 (30%) presented seropositivity to IgG anti-*Toxoplasma* (Figure 5).

### 3.3. Analysis of Studies on Subgroup Prevalence by Microorganism Type

**Figure 6:**
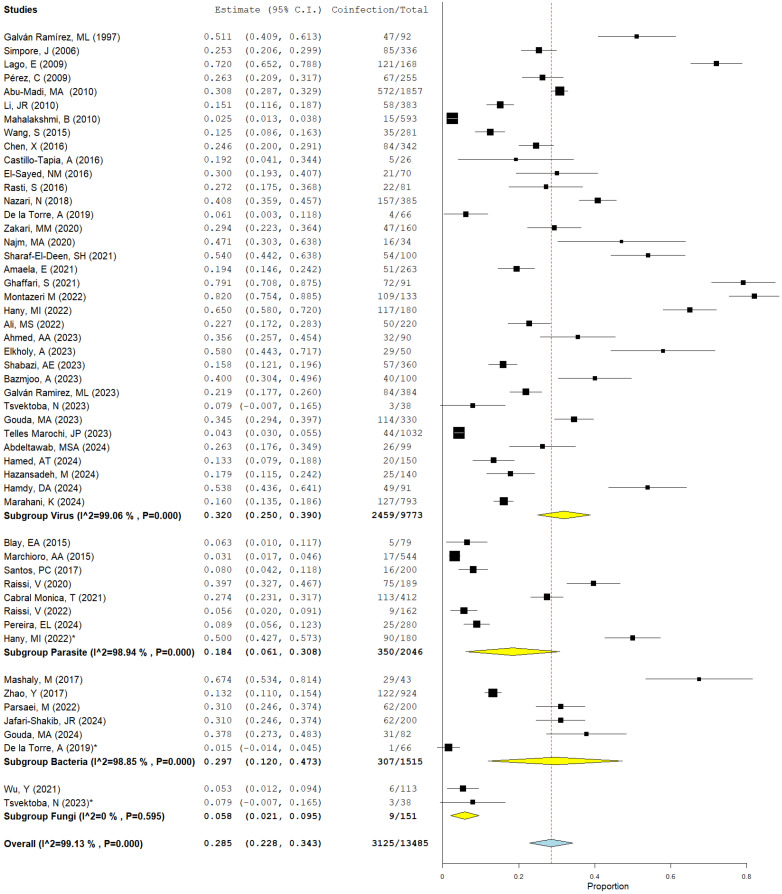
Forest plot of * studies of multiple coinfections. The black squares show size effects, and the lines represent confidence intervals (95% C.I.) Yellow diamonds indicate the prevalence of *Toxoplasma* coinfection for each type of microorganism; the blue diamond indicates pooled prevalence, I^2=Heretogenity,, (references [10,11,12,13,14,15,16,17,18,19,20,21,22,23,24,25,26,27,28,29,30,31,32,33,34,35,36,37,38,39,40,41,42,43,44,45,46,47,48,49,50,51,52,53,54,55,56,57]).

The prevalence of coinfection was highest in the subgroups for viruses (32.0%, I^2^ 99.06%, *p* < 0.0001), and bacteria (29.7%, I^2^ 98.85%, *p* < 0.0001) followed by the subgroup for parasites (18.4%, I^2^ 98.94%, *p* < 0.0001) and finally, the subgroup for fungi (5.8%, I^2^ 0%, *p* < 0.595). The black squares show size effects, and the lines represent confidence intervals Figure 6.

## 4. Discussion

Toxoplasmosis is a highly prevalent disease. Its wide range is due to various factors; these include eating habits, ages of populations, migration patterns, and the coexistence of humans with cats [1,4].

*Toxoplasma gondii* is an opportunist protozoan that mainly affects populations with some immunocompromised individuals. In the present review, we found that *Toxoplasma gondii* was most closely associated with viral infections, with HIV ranking first, followed by cytomegalovirus, hepatitis B and C, rubella, herpes simplex 1 and 2, SARS-CoV-2, and coxsackievirus.

The results of our review show significantly more articles related to the coinfection of *Toxoplasma gondii* with HIV than with other pathogens; a total of 936 coinfected patients were described in these studies [10,11,12,13,15,20,21,27,30,34,49,57]. Such infections are due to immunosuppression characterized by a decrease in the CD4+ T cell count [1,3] and inflammatory cytokines such as interferon γ and interleukins IL-6, and IL-18 [1].

Coinfection with SARS-CoV-2 was reported in a number of studies, involving a total of 701 patients [32,35,38,41,42,43,44,46,48,50,53,56]. Both conditions may stimulate the immune system by activating toll-like receptors (TLR2, TLR4, and TLR7) [32]. Some cytokines present in patients with toxoplasmosis could increase the severity of the symptoms of COVID-19 [32,46]. On the other hand, we found no report which showed any positive association between SARS-CoV-2 and *T. gondii.*

In this study, Egypt, Iran, and Iraq had the most cases of coinfection with toxoplasmosis and SARS-CoV-2. However, according to the World Health Organization, the Americas have been most affected by SARS-CoV-2 to date. In light of the fact that more than 775 million confirmed cases of COVID-19 had been identified by June 2024, it is likely that increased numbers of cases of coinfection with toxoplasmosis and SARS-CoV-2 will be identified in future studies.

In our review, we found three articles related to *Toxoplasma*, *Rubella*, *Cytomegalovirus,* and Herpes (TORCH) infections in newborns. In this group, coinfection with *T. gondii* and CMV was found to be most prevalent [14,16,23]. This may have been due to a higher rate of maternal transmission than that recorded for either HSV, which is usually infrequent in neonates, or rubella, which now has a lower rate of incidence as a result of vaccination programs and improved maternal–fetal care [16]. There is an established association between poor prenatal care and TORCH infections during pregnancy. In light of this, women of gestational age should take precautions to avoid infection with *Toxoplasma gondii* infection, in addition to other microorganisms. Such precautions should include proper handwashing before and after handling meat, only eating well-cooked meat, wearing gloves when gardening, and avoiding contact with cat feces [1,3].

Coinfection with bacteria was reported in six population studies, revealing an overall prevalence of 29.7%; of these studies, four involved tuberculosis and *Toxoplasma gondii* [24,26,37,54]. In recent years, coinfection with *T. gondii* and TB has become an emerging public health problem in developing countries. During active TB infection, there is a decrease in the production of Th1 response cytokines, and an overproduction of Th2 cytokines. Consequently, the abrupt change in immune response and the subsequent suppression of cell-mediated immunity against toxoplasmosis can reactivate or increase susceptibility to a new infection [24].

Since 2013, the number of tuberculosis cases has remained the same as it was at the beginning of the present century. According to the WHO, there are three million new cases of TB each year, many of which go unnoticed. Recently, the WHO called for accurate diagnosis of tuberculosis caused by *M. bovis* in humans. It is also important to mention that if a person has an active disease, and that person has not been diagnosed and treated, they become a source of contagion in their community. Because of this transmission mechanism, TB is a difficult-to-manage disease; in addition, the immunological weaknesses caused by TB mean that patients with the disease face a high risk of contracting other infections.

Tuberculosis is named on the WHO list of the most important zoonotic diseases worldwide because a quarter of the global population has been in contact with this bacterium, and approximately 10% have developed the disease. Furthermore, the population most at risk is the immunocompromised. For example, HIV patients face a 16-fold greater risk of developing active TB. In our results, cases of coinfection with toxoplasmosis and TB were most prevalent in Congo, Nigeria, India, China, and Indonesia. These countries are the most affected because two-thirds of the world’s cases of TB were recorded amongst their national populations.

*T. gondii* infection in patients with tuberculosis is also strongly related to contact with the feces of both domestic and stray cats, and an association with the consumption of raw meat has also been reported [1,24,54].

In the present review, coinfection with parasites and toxoplasmosis most frequently involved *Toxocara*; this was referenced in 6 studies involving a total of 255 coinfected patients [18,25,29,33,40,51]. However, it is the case that coinfection with *T. gondii* and *Toxocara* spp. is usually less noticeable in emerging countries, though still frequent among women and children. The zoonoses of *T. gondii* and *Toxocara* spp. are both highly prevalent, bringing potentially serious long-term consequences for infected individuals. These infections involve similar risk factors, and both are associated with high costs for healthcare institutions. A study of the risks and costs associated with these parasites conducted at the Canadian Institute for Health Information (2002–2011) found an annual incidence of severe toxoplasmosis and toxocariasis of 0.257 (95% confidence interval [CI]: 0.254–0.260) cases per 100,000 people.

In toxocariasis, mammals (most importantly, dogs and cats) ingest the eggs from the soil or become infected by eating the meat of infected animals. The worms complete their life cycle in the infected host, and the eggs are shed in the feces. Toxocariasis is transmitted to humans by the ingestion of eggs from the soil or from contaminated hands, food, or formites [1,3,51]. *Toxocara* can increase the severity and incidence of intracellular parasitic infections by increasing the Th2-type response and decreasing the Th1-type response. In addition, several risk factors are related to the acquisition and even reactivation of *Toxoplasma gondii* infection; among these, we may mention lifestyle, socioeconomic, and cultural factors [5], as well as levels of education [27], contact with animals that are carriers of the parasite, transplants, and situations in which patients are immunocompromised and have had blood transfusions.

We found only two articles related to coinfection with fungi, i.e., a prevalence of 5.8%. This may have been due to the low number of relevant studies included. In addition, several risk factors are related to the acquisition and even reactivation of *Toxoplasma gondii* infection; among these are lifestyle, socioeconomic and educational status, and cultural factors [5,27], as well as contact with animals that are carriers of the parasite and the recipients of donated organs and blood transfusions [1].

ELISA was the principal method used to diagnose toxoplasmosis; however, other methods were applied, depending on the clinical status and characteristics of patients. For example, computer tomography (TC), Chemiluminescence (CMIA), and enzyme-linked fluorescent assay (ELFA) were used.

Zoonoses are a public health problem that affects a large part of the world’s population. In this study, *Toxocara*, *Plasmodium*, and TB were all considered zoonotic diseases. These diseases involve more complex means of control because they affect both human and animal health. This means that efforts must be made in different health disciplines if effective control is to be achieved.

Due to the high impact of toxoplasmosis, it is necessary to consider a series of preventive and control actions among populations worldwide. Prevention programs should communicate the following guidelines: (1) meat should be cooked sufficiently well to prevent contamination by cysts; (2) vegetables that can be contaminated with oocysts; (3) removal of cat feces and subsequent cleaning of affected areas should be carried out using gloves and after-hand washing; (4) all fruit should be washed before eating; and (5) pregnant women should seek information from their doctor regarding taking and anti-*Toxoplasma* antibody test. Adoption of these measures should reduce the high prevalence of infection and complications in immunocompromised patients. More generally, the better the implementation of prevention and control programs by health institutions worldwide, the more they can help reduce the economic costs caused by toxoplasmosis in the world’s population.

Finally, the results reported in the 48 articles included in our review showed that most infections were chronic because patients only had IgG antibodies. The 11 studies of acute toxoplasmosis where patients had symptoms and positive IgM all corresponded to studies of viral infections such as HIV, SARS-CoV-2, HSV, and CMV. The IgM antibodies were less prevalent and in immunocompromised patients were mainly not detected, which is in accordance with previous reports in the literature [10,11]. This may have been because these patients’ immunocompromised status made them more susceptible to new or reactive infections of *T. gondii*. Other studies that indicated cerebral toxoplasmosis were confirmed by computed axial tomography.

## 5. Conclusions

Confections with *Toxoplasma* and human immunodeficiency virus (HIV) were most frequently reported.

The coinfections of *T. gondii* with parasites were less frequently reported, but strong associations with *Toxocara* were identified.

The most reported coinfection of *T. gondii* with *Mycobacterium* was in cases of *tuberculosis.*

The findings of the meta-analysis presented in this paper may help physicians, epidemiologists, infectious disease specialists, and researchers interested in parasitology.

## Figures and Tables

**Table 1:** Publications included in the systematic review.

Number of Article Included	Year	First Author	Country	Average Age (Years)	Range (Years)	Type of Study	No. of Subjects	Ref.
1	1997	Galván-Ramírez, M.L.	Mexico	34	17–69	CS	92	[10]
2	2006	Simpore, J.	Burkina Faso	NS	18–45	CS	336	[11]
3	2009	Lago, E.	Brazil	27.1	NS	CS	2267	[12]
4	2009	Pérez, C.	Chile	40.9	NS	CS	225	[13]
5	2010	Abu-Madi, M.A.	Qatar	NS	<6 mo to 45 y	CS	1857	[14]
6	2010	Li, J.R.	China	30.9	18–58	CC	439	[15]
7	2010	Mahalakshmi, B.	India	NS	10 d to 12 mo	CS	593	[16]
8	2015	Blay, E.A.	Ghana	28	18–42	CS	79	[17]
9	2015	Marchioro, A.A.	Brazil	NS	0–12	CS	544	[18]
10	2015	Wang, S.	China	NS	0–3	CC	503	[19]
11	2016	Chen, X.	China	31	13–68	CS	1414	[20]
12	2016	Castillo-Tapia, A.	México	NS	3–16	CS	26	[21]
13	2016	El-Sayed, N.M.	Egypt	NS	19–66	CC	120	[22]
14	2016	Rasti, S.	Iran	C 28.2	NS	CC	179	[23]
				CTR 28.6				
15	2017	Mashaly, M.	Egypt	C 40.9	NS	CS	330	[24]
				CTR 37.1				
16	2017	Santos, P.C.	Brazil	NS	NS	CS	280	[25]
17	2017	Zhao, Y.	China	NS	30–60	CC	1848	[26]
18	2018	Nazari, N.	Iran	35.95	3–68	CS	385	[27]
19	2019	De-la-Torre, A.	Colombia	37.5	13–70	CS	66	[28]
20	2020	Raissi V.	Iran	30.2	18 to >39	CC	378	[29]
21	2020	Zakari, M.M.	Nigeria	NS	19–40	CS	320	[30]
22	2020	Najm, M.A.	Iraq	NS	>20 to 41	CC	68	[31]
23	2021	Sharaf-El-Deen, S.H.	Egypt	C > 60	NS	CC	150	[32]
				CTR < 60				
24	2021	Cabral Monica, T.	Brazil	NS	4–15	CS	412	[33]
25	2021	Amaela, E.	Rep. Congo	42.6	16–71	CS	263	[34]
26	2021	Ghaffari, S.	Iran	60.9	9–90	CC	269	[35]
27	2021	Wu, Y.	China	NS	NS	CS	113	[36]
28	2022	Parsaei, M.	Iran	40.6	NS	CC	200	[37]
29	2022	Montazeri, M.	Iran	58.9	NS	CS	133	[38]
30	2022	Hany, M.I.	Egypt	50.3	21–70	CS	180	[39]
31	2022	Raissi, V.	Iran	NS	20–50	CC	162	[40]
32	2022	Ali, M.S.	Iraq	NS	20–60	CS	220	[41]
33	2023	Ahmed, A.A.	Iraq	NS	10–70	CS	120	[42]
34	2023	Elkholy, A.	Egypt	52.2	NS	CS	50	[43]
35	2023	Shabazi, A.E.	Iran	NS	1–60	CS	360	[44]
36	2023	Bazmjoo, A.	Iran	43.79	NS	CS	100	[45]
37	2023	Galvan-Ramirez M.L.	Mexico	47.3	19–74	CS	384	[46]
38	2023	Tsvektoba, N.	Bulgaria	NS	1–18	CS	38	[47]
39	2023	Gouda, M.A.	Egypt	47.69	14–78	CS	330	[48]
40	2023	Telles Marochi, J.P.	Brazil	44	35–50	CS	1032	[49]
41	2024	Abdeltawab, M.S.A.	Egypt	C 56.4	NS	CS	100	[50]
				CTR 56.3				
42	2024	Pereira, E.L.	Brazil	26	15–43	CS	280	[51]
43	2024	Hamed, A.T.	Iraq	NS	NS	CC	150	[52]
44	2024	Hazansadeh, M.	Iraq	C 36	NS	CC	140	[53]
				CTR 37				
45	2024	Jafari-Shakib, R.	Iran	NS	NS	CC	200	[54]
46	2024	Gouda, M.A.	Egypt	33.68	25–47	CS	82	[55]
47	2024	Hamdy, D.A.	Egypt	C 43.5	20–80	CS	91	[56]
				CTR 44				
48	2024	Marahani, K.	Indonesia	HIV+ 36	NS	CS	793	[57]
				HIV− 22				
**Total**							**17,535**	

CS—cross-sectional; CC—case and controls; C—cases; CTR—controls; NS—no specific month (mo) or day (d).

**Table 2:** Prevalence of anti-*Toxoplasma* IgG/IgM antibodies, symptoms, and treatments.

Number of Article Included	Year	First Author	Symp	Type of Microorganism	Method Diagnostic	No. of Subjects	IgG	IgM	No. COIN	Treatment	Ref.
1	1997	Galván-Ramírez, M.L.	3	HIV	ELISA	92	46	1	47	NS	[10]
2	2006	Simpore, J.	3	HIV	ELISA	336	85	ND	85	NS	[11]
3	2009	Lago, E.	3	HIV	ELFA	2267	1745	ND	121	ART	[12]
4	2009	Pérez, C.	3	HIV	ELISA	225	67	NS	67	NS	[13]
5	2010	Abu-Madi, M.A.	1	*Rubella*, CMV, HSV	ELISA	1857	378	16	76	NS	[14]
6	2010	Li, J.R.	2	HIV	ELISA	439	69	ND	58	NS	[15]
7	2010	Mahalakshmi, B.	3	*Rubella*, CMV, HSV	ELISA	593	50	7	15	NS	[16]
8	2015	Blay, E.A.	3	*Plasmodium falciparum*	PCR	79	60	ND	5	NS	[17]
9	2015	Marchioro, A.A.	3	*Toxocara* spp.	ELISA-IF	544	40	NS	17	NS	[18]
10	2015	Wang, S.	3	*Coxsackie* A16	ELISA	503	35	NS	35	NS	[19]
11	2016	Chen, X.	3	HIV	ELISA	1414	447	NS	84	NS	[20]
12	2016	Castillo-Tapia, A	3	HIV	ELISA	26	5	ND	5	*	[21]
13	2016	El-Sayed, N.M.	3	HBV, HCV	PCR	120	24	ND	21	NS	[22]
14	2016	Rasti, S.	1	*Rubella*, CMV, HSV	ELISA	179	50	1 C	22	NS	[23]
								2 CTR			
15	2017	Mashaly, M.	3	*M. tuberculosis*	ELISA	330	42	UN	29	RIF	[24]
								UN			
16	2017	Santos, P.C.	3	*Toxocara* spp.	ELISA	280	125	UN	16	NS	[25]
17	2017	Zhao, Y.	3	*M. tuberculosis*	ELISA	1848	212	19	122	NS	[26]
18	2018	Nazari, N.	3	HIV	ELISA	385	157	10	157	ART, CTX, AZM	[27]
19	2019	De-la-Torre, A.	1	VZV, CMV, *M. tuberculosis*	ELISA-PCR	66	22	ND	5	VAL, ATX	[28]
20	2020	Raissi V.	3	*Toxocara* spp.	ELISA	378	94	ND	75	NS	[29]
21	2020	Zakari, M.M.	3	HIV	ELISA	320	92	12	47	NS	[30]
22	2020	Najm, M.A.	3	*Rubella*	PCR	68	30	19 C	16	NS	[31]
								11 CTR			
23	2021	Sharaf-El-Deen, S.H.	3	SARS-CoV-2	ELISA	150	72	UN	54	NS	[32]
24	2021	Cabral Monica, T.	3	*Toxocara canis*	ELISA	412	234	UN	113	NS	[33]
25	2021	Amaela, E.	1	HIV	CT	263	51	ND	51	CTX, ART	[34]
26	2021	Ghaffari, S.	1	SARS-CoV-2	ELISA	269	226	UN	72	NS	[35]
27	2021	Wu, Y.	3	*P. jirovecii*	DUPLEXqPCR	113	3	UN	6	NS	[36]
28	2022	Parsaei, M.	3	*M. tuberculosis*	ELISA-nestPCR	200	95	UN	62	NS	[37]
29	2022	Montazeri, M.	3	SARS-CoV-2	ELISA-PCR	133	109	ND	109	NS	[38]
30	2022	Hany, M.I.	3	*E.histolytica*, *S. mansoni*, HCV	ELISA	180	117	7	41	NS	[39]
31	2022	Raissi, V.	3	*Toxocara* spp.	ELISA	162	31	UN	9	NS	[40]
32	2022	Ali, M.S.	3	SARS-CoV-2	ELISA	220	75	2	50	NS	[41]
33	2022	Ahmed, A.A.	3	SARS-CoV-2	ELISA	120	30	2	32	NS	[42]
34	2023	Elkholy, A.	3	SARS-CoV-2	ELISA	50	29	ND	29	NS	[43]
35	2023	Shabazi, A.E.	3	SARS-CoV-2	ELISA	360	103	UN	57	NS	[44]
36	2023	Bazmjoo, A.	3	HBV, HCV	ELISA-PCR	100	22	ND	40	NS	[45]
37	2023	Galvan-Ramirez M.L.	3	SARS-CoV-2	ELISA	384	105	26	84	NS	[46]
38	2023	Tsvektoba, N.	3	*P. jirovecii*	PCR	38	3	NR	3	NS	[47]
39	2023	Gouda, M.A.	3	SARS-CoV-2	ELISA-PCR	330	114	ND	114	NS	[48]
40	2023	Telles Marochi, J.P.	1	HIV	ELISA	1032	44	UN	44	TMP/SMX, PYR, ART	[49]
41	2024	Abdeltawab, M.S.A.	3	SARS-CoV-2	ELISA-PCR	100	31	UN	26	NS	[50]
							C LowA 15				
							C HighA 10				
							CTR LowA 4				
							CTR HighA 1				
42	2024	Pereira, E.L.	3	*Toxocara* spp.	CMIA	280	69	UN	25	NS	[51]
43	2024	Hamed, A.T.	3	HCV	ELISA	150	20	NS	20	NS	[52]
44	2024	Hazansadeh, M.	2	SARS-CoV-2	ELISA	140	44	ND	25	NS	[53]
45	2024	Jafari-Shakib, R.	3	*M. tuberculosis*	ELISA	200	132	2	62	NS	[54]
46	2024	Gouda, M.A.	2	*H. pylori*	ELISA	82	35	UN	31	NS	[55]
47	2024	Hamdy, D.A.	3	SARS-CoV-2	ELISA	91	52	UN	49	NS	[56]
48	2024	Marahani, K.	1	HIV	CT	793	127	ND	127	ART	[57]
Total					17,535		5848	125	2460		

Symp—symptoms; COIN—coinfection with *Toxoplasma* and other microorganisms; HIV—human immunodeficiency virus (*HIV*); CMV—c*ytomegalovirus*; HSV—*herpes simplex virus*; HBV—h*epatitis B virus*; HCV—h*epatitis C virus*; VZV—v*aricella zoster virus*; SARS-CoV-2—s*evere acute respiratory syndrome*; *E. histolytica*—*Entamoeba histolytica*; *S. mansoni*—*Schistosoma mansoni*; *P. jirovecii*—*Pneumocystis jirovecii*; *H. pylori*—*Helicobacter pylori*; CS—cross-sectional; CC—case and controls; ELISA—enzyme-linked immunoassay (ELISA); ELFA—enzyme-linked fluorescent assay; PCR—polymerase chain reaction; IF—indirect immunofluorescence (IF); CT—computed tomography; CMIA—chemiluminescence microparticle immune assay. Symptoms: (1) symptomatic; (2) asymptomatic; (3) not specific (NS). not detectable (ND), unrealized (UN), C—cases; CTR—controls; *Zidovudine (AZT), *Lamivudine (3TC), *Lopinavir (LPV), *Lopinavir/ritonavir (LPV/r), *Efavirenz (EFV), *TDF = Tenofovir (TDF), *Emtricitabine (FTC), *Didanosine (ddl), *RAL = Raltegravir (RAL), *DRV = Darunavir (DRV), *Etravirine (ETR), *Selzentry (MVC), AZT—zidovudine; 3TC—lamivudine; LPV—lopinavir; LPV/r—lopinavir/ritonavir; EFV—efavirenz; TDF—tenofovir; FTC—emtricitabine; ddl—didanosine; RAL—raltegravir; DRV—darunavir; ETR—etravirine; MVC—selzentry; RIF—rifampicin; ART—antiretroviral therapy; CTX—co-trimoxazole; AZM—szithromycin; VAL—valacyclovir; ATX—anti-*Toxoplasma* therapy; TMP/SMX—trimethoprim sulfamethoxazole (TMP/SMX); PYR—pyrimethamine; LowA—low avidity; HighA—high avidity.

## Data Availability

The original contributions presented in the study are included in the article, further inquiries can be directed to the corresponding author.
